# Royal Medical and Chirurgical Society — an Analysis of One Hundred Cases of Cancerous Disease of the Uterus

**Published:** 1852-09

**Authors:** 


*Royal Medical and Chirurgical Society — An Analysis of One Hundred Cases of Cancerous Disease of the Uterus.* By Robert Lee, M. D., F. R. S. &c.

The conclusions to which the author arrived from this analysis were:—1st. That cancer may commence in any part of the mucous, muscular, or peritoneal coats of the uterus; but most frequently in the os and cervix. 2dly. That the earliest symptoms of the disease, in a large proportion of cases, were discharges of sanguineous, serous, or white-colored fluid from the vagina, with sense of uneasiness or pain more or less acute within and around the pelvis. 3dly. That cancerous disease of the uterus presented itself most frequently in the form of induration and ulceration of the os and cervix uteri and vagina, or ulceration without induration, or in the form of fungoid tumors, usually called cauliflower excrescences, growing from one of the lips or the whole os uteri, being often associated with encephaloid or colloid masses and true scirrhus of the remaining portions of the uterus and contiguous viscera. 4thly. That in no case could cancerous disease of the uterus be referred to inflammation; and that its fatal progress was never arrested by cauterizing the morbid structures through the speculum, nor by any other course of treatment.—*Dublin Med. Press.*

